# No strings attached: physiological monitoring of rhesus monkeys *(Macaca mulatta)* with thermal imaging

**DOI:** 10.3389/fnbeh.2015.00160

**Published:** 2015-06-19

**Authors:** Stephanos Ioannou, Hélène Chotard, Marina Davila-Ross

**Affiliations:** ^1^Department of Neuroscience, Section of Physiology, University of ParmaParma, Italy; ^2^Department of Psychology, University of PortsmouthPortsmouth, UK

**Keywords:** thermal imaging, physiology, rhesus macaques, emotions, autonomic nervous system, temperature

## Abstract

Methodological challenges make physiological affective observations very restrictive as in many cases they take place in a laboratory setting rather than the animals' natural habitat. In the current study using Infrared Thermal Imaging we examine the physiological thermal imprints of five macaques. The monkeys were exposed in three different experimental scenarios. Playing with a toy, food teasing as well as feeding. It was observed that during teasing the temperature of the region surrounding the eyes was higher than play as a result of rapid saccades directed at the food. Compared to play and teasing, a lower temperature accompanied feeding on the upper lip, nose and orbital region suggesting elevated levels of distress. These findings prove that thermal imaging is a reliable method of physiological monitoring the subject at a distance while preserving a semi-experimental setting.

## Introduction

Emotional experiences are tuned by the autonomic nervous system (ANS) preparing us to face a plethora of environmental challenges (Levenson, 2003). Hair erection, vascular constriction, perspiration, heart rate, respiration as well as the inhibition of the digestive tract have their own biological function in survival (Porges, 2001). Each emotion is driven by characteristic physiological elements and in the majority of cases conscious awareness of the somatic sensation gives feedback for avoidance or engagement (Damasio, 1996). Autonomic excitation precedes behavioral engagement and by cognitively evaluating the situation in which the physiological episode arose; descriptive labels are commonly attributed to the felt emotion (Schachter and Singer, 1962; Dutton and Aron, 1974). Although in humans a somatosensory distinction between the different types of emotion exists (Nummenmaa et al., 2014), for non-human primates one has to draw conclusions either by coding behaviors or by assessing endocrinological, skeletomuscular, or neurological changes (Levenson, 2003).

Conventional physiological measurements limit the way biological data is collected since they require restriction of the subject's movement (Nakayama et al., 2005; Kuraoka and Nakamura, 2011) or invasive implantation of radio telemetric probes for autonomic monitoring (Vianna and Carrive, 2005). Direct contact with the body limits the nature of the experimental paradigm and the use of temperature sensors on the skin is not an option as they get detached through contact, cover a small surface area and pressure on the skin can induce changes to regional blood flow affecting physiological recordings (Nakayama et al., 2005). Functional Infrared Thermal Imaging (fITI) is a highly sensitive and versatile technique that converts infrared light into temperature allowing wireless monitoring of the participant (Ring and Ammer, 2012).

So far the non-human primate studies that used thermal imaging used stress-inducing experimental paradigms. Nakayama et al. (2005) after exposing macaques to a threatening person observed the nose temperature consistently going down. In addition, transient increases in temperature at the peri-orbital region were also recorded, although inconsistent among subjects (Nakayama et al., 2005). Eye temperature increases have been observed in cows during stress (Stewart et al., 2007). In another study involving macaques, subjects exposed to videos with aggressive threats or screams showed a temperature decrease on the nose. Electrophysiological recording that used the same threatening stimuli demonstrated increased neuronal activity in the monkey's amygdala. Similar results on the nose tip have been obtained in humans in negative emotional states (Ioannou et al., 2013). Although the above non-human primate studies represent a first step toward understanding facial skin temperatures in species closely related to humans, they hardly represent a naturalistic experimental setting.

The current study was set to examine with fITI how macaques react to playing, teasing and feeding while retaining a semi-experimental setting. Unlike previous studies in thermal physiology, temperature extraction was based on four different facial regions of interest the nose bridge and tip, the maxillary area and the peri-orbital region. The main comparison of the current study was between teasing (negative), play (positive), and feeding. These experimental categories aim at eliciting physiological changes that reflect the two competing subdivisions of the ANS the sympathetic (food teasing) and para-sympathetic (play and feeding). It is important to note however, that a true comparison between the opposing subdivision of the ANS hardly exists as empirical evidence show that the parasympathetic nervous system plays a role in both pleasant and unpleasant emotions (Kreibig, 2010, p. 408). Experimental conditions that represent a “baseline” or “a perfect contrast pair” have always been a major methodological challenge in physiology (Levenson, 1988), a task that gets even more difficult when dealing with non-human subjects. Thus, with the current experimental conditions we aim to create with the chosen experimental conditions adequate physiological contrast to describe the directionality of the thermal change and explore the plausibility of harnessing thermal prints from non-restrained subjects.

Although temperature related animal literature is scarce, human studies that exposed participants in real-life “harassment” scenarios (eliciting anger) have observed an increase in forehead temperature, α-adrenergic, and β-adrenergic increases, local increases in facial blood perfusion (Stemmler et al., 1991) and vascular constriction on facial periphery (Prkachin et al., 1999). Thus, it is expected that teasing will lead to elevated temperature patterns on the peri-orbital region, as the supraorbital branch that innervates the forehead and the superior palpebral branch are thermally coherent (Pavlidis et al., 2002; Ioannou et al., 2014a). During this experimental phase chollinergic and β-adrenergic sympathetic stimulation of the blood vessels could account for small vasodilatory effects on specific facial regions (Smith and Kampine, 1990). On the other hand playing with a toy aimed at arousing feelings of amusement or happiness in the subjects. Whereas amusement is characterized by vagal engagement and a α-adrenergic increase happiness is characterized by vagal withdrawal (Van Reekum et al., 2004). Nevertheless, in both occasions it is believed that this will lead to a temperature decrease on to the periphery of the face as a result of α-adrenergic influence since vascular constriction independent of heart rate defines the emitted heat signals (Vianna and Carrive, 2005). Feeding has been added as an additional condition to examine since in many biology textbooks the parasympathetic nervous system has been characterized as the “resting and digestive” system (Herlihy, 2015, p. 220). In contrary to autonomic arousal during a feeding episode the 10th cranial nerve that controls the heart also innervates sub-diaphragmatic visceral organs relaxing the gut in order to promote digestive processes. This system which has been also named dorsal vagal complex (Porges, 2001) is associated with bradycardia and vasodilation thus one would expect during this phase for temperature on the face to rise. Lastly behavioral analysis was also conducted to ensure that temperature changes for each experimental condition did not result from the subjects' movement. Observed temperature changes throughout the conditions would suggest that animals could be physiologically monitored from a distance with fITI providing new insights in biological research. The aim of this study and the current article is to pave the way for a new promising experimental method in the field of behavioral neuroscience.

## Materials and methods

### Subjects and study site

Subjects were five rhesus macaques, 3 males (1 juvenile and 2 adults), and 2 females (1 juvenile and 1 adult) at the Owl and Monkey Haven (Isle of Wight, UK). Together they represented one social group, living in an outdoor enclosure during the day and in an indoor enclosure during the night. Testing took place at the outdoor enclosure, which was surrounded by a mesh with about 35 × 35 mm hole sizes and 3 mm wire thickness. The outdoor enclosure contained climbing structures, ropes, plastics bowls and the ground was covered with small stones. The macaques were fed at 8 a.m., 3 p.m. and 5 p.m. and had water available throughout the day and night.

### Procedure

**Figure 1:**
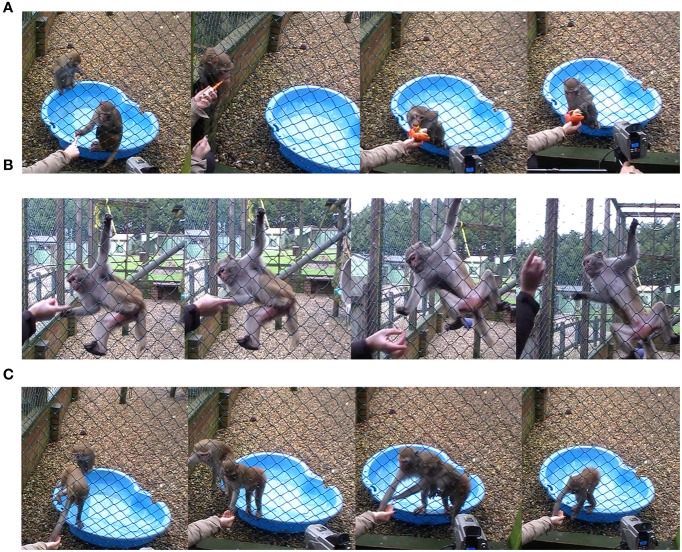
**Illustration of the three experimental session and the behaviors that were exhibited by the subjects (A) Playing (B) Teasing with food (C) Feeding**.

Play, teasing, and feeding sessions were induced. For play, the experimenter presented a toy (a baby rattle, a cuddly toy, or a doll purse) to the subject and performed playful up-and-down movements to evoke play responses. If a subject did not respond to a particular toy, another was presented. For teasing, the experimenter held food (mealworm or cricket) and moved the food slowly toward the subject and quickly backs behind the mesh, so that the subject could not reach it. For feeding, the experimenter gave the subject one mealworm or cricket. Throughout the testing, the experimenter drew the attention of only one subject at a time and made it stay close to the mesh throughout the test. However, despite this previous step, others individuals were present next to the subject during 14 play sessions and 16 teasing as well as feeding sessions. Figure 1 provides an illustration of the experimental sessions and the behaviors that were exhibited by the subjects.

Each play and teasing session was aimed to last 60 s; some sessions lasted shorter (minimum of 15 s), when a subject lost interest earlier or when another macaque interfered. Furthermore, teasing was stopped as soon as the subject showed signs of being upset (e.g., bark) and lasted approximately 2 min. Play sessions lasted longer (up to 4 min) when it took longer for the subjects to show some interest in the toy or to show play responses. The feeding sessions lasted between 15 and 25 s. In all experimental conditions if the data for a particular subject was not long enough to be included in the analyses the experimenter re-tried until the subject started to get more interested on the stimulus. If all efforts failed the data for the non-interested subject had to be discarded and the testing had to take place another day for this subject.

### Data collection

For the thermal recordings, a TP8 Camera (ThermoPro™) was used with an acquisition rate of 1 frame per second, a resolution of 384 × 288 pixels, a sensitivity of 0.08 K, and an accuracy of ±0.1°C. It was placed approximately one meter away and in front of the subject aiming at capturing frontal shots and recorded under no direct sunlight. In addition, the subjects were simultaneously video-recorded with a regular camcorder (JVC Everio). The experimenter stood no further than 1 meter away from the opposite sides of the enclosure mesh. Altogether 26 play, 16 teasing, and 30 feeding sessions were recorded on 2 days, in November 2013 and January 2014. For both days the test sessions started off with playing and feeding, followed by teasing. Between each test session a period of up to 5 min was allowed in order for the subjects face to return back to baseline values during which the experimenter was out of sight. No high-arousal interactions (such as fights or sexual behaviors) were observed during the testing and breaks. In order to collect an adequate amount of data in total four sessions took place, two for each day. The outdoor temperatures and the hours of the day were reported for play (mean: 6.5°C; range: 10:30 a.m. to 2 p.m.), teasing (6.0°C; 2 p.m. to 3 p.m.) and feeding (6.5°C; 10:30 a.m. to 2 p.m.).

### Thermal data analyses

**Figure 2:**
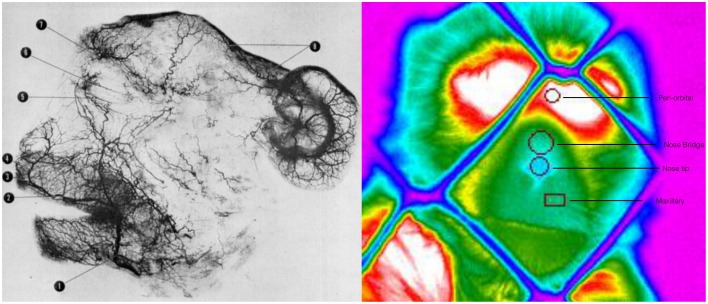
**Arteriograph representing the arterial vessels of face of a rhesus monkey Facial artery (1); superior labial (2); septal branch (3); lateral nasal branch (4); inferior palpebral branch (5); superior palpebral branch (6); supraorbital branch (7); parietal and frontal branches (8) (Teichmann's paste injection, cleared preparation) (Castelli and Huelke, 1965)**. Thermogram representing the regions of interest, their positioning and the selected shape for temperature extraction.

Temperature frames were extracted for each individual every 5 s. The criteria for frame extraction were that the subject's face ought to be facing the camera or be homogenous in angle (no more than a 45° in angle) across the extracted frames. In addition the mesh should not heavily obscure the regions of interest. Temperature data were extracted from four regions of interest based on the anatomical facial artery system of rhesus monkeys provided by Castelli and Huelke (1965). Circular extraction points were placed on the nose bridge and tip, horizontally and superior of the lateral nasal arterial branch, a rectangular extraction shape was placed on the maxillary area, on the septal arterial brunch, and a circular region of interest on the the peri-orbital region medially between the inferior and superior palpebral brunch (see Figure 2). In extreme occasions where a region of interest was obscured by the mesh the next available frame was used. This does not affect the main statistical analyses as the development of the thermal signal takes at the best-case scenario 10 s to significantly change (Ioannou et al., 2014a). Stem and leaf plots were conducted prior to the main analyses to eliminate outliers across individuals. All frames were extracted by an individual naïve to the experimental protocol and afterwards an experienced thermographer examined all the frames regarding the positioning of the regions of interest. In total 1024 temperature data points were extracted for analyses. To decide upon the main statistical analyses, conditions were examined for intrinsic inter-correlations using Spearman's rho (Nakayama et al., 2005). Although ideally we would like for our predictors (independent) variables to be strongly related to our dependent variable we would not want our conditions to be related. If they are not related a 1 × 3 within-conditions ANOVA can be performed comparing each individual condition with each other based on the corresponding region of interest. However, in the case that they are related (correlated) such as feeding and teasing for the maxillary area (*r_s_* = 0.9, *p* < 0.05, *n* = 5) and feeding and playing for the nose tip (*r_s_* = 0.9, *p* < 0.05, *n* = 5) a more conservative approach should be followed. Orthogonal contrasts protect against autocorrelation and are an excellent way of analyzing data to obtain main effects and comparisons between groups of means (Doncaster and Davey, 2007). Contrasts involve linear combinations of group mean vectors instead of linear combinations of the variables. For example in the case of the current experiment the first two conditions playing and teasing form the first contrast dyad, whereas the second contrast in this case feeding is compared with the previous two conditions (Paying and Teasing) forming the second and last comparison of the ANOVA. The same approach was followed in Ioannou et al. (2013). For each macaque the mean temperature value of each region of interest was calculated (nose tip, maxillary, periorbital, nose bridge) according to condition (playing, teasing, feeding). Then a 1 × 3 repeated measures ANOVA contrast was conducted separately for each of the four regions of interest (3 conditions × 5 subjects). Mann–Whitney *U*-tests were also performed to assess for every subject if the facial temperatures significantly differed across the conditions using orthogonal contrasts. These test results were then used to evaluate how many subjects showed temperature changes that matched the group results. So for example in order to observe if all five subjects during the feeding phase had a temperature decrease a Mann–Whitney *U*-test was performed. This was conducted for every region of interest for each individual subject.

### Behavioral coding

Behavioral data were obtained to control for temperature artifacts that may have resulted by changes in the intensity of movement and not by the applied experimental manipulation. Bodily movements were coded as being either slow (e.g., touching, licking, and smelling the toy during play; slowly reaching out for the food during teasing) or rapid (e.g., repetitive grabbing directed toward food during teasing). They were coded each second per session and then added to the extracted thermal values with identical times and sessions. The movements were coded by one researcher, who was inter-coder reliability tested (Cohen's Kappa, *K* = 0.74; for 15 sessions, 5 subjects).

## Results

### Group temperature in relation to condition

**Figure 3:**
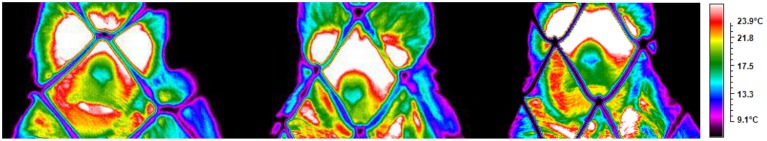
**Illustration from a subject depicting the temperature across conditions (playing, teasing, feeding)**.

**Figure 4:**
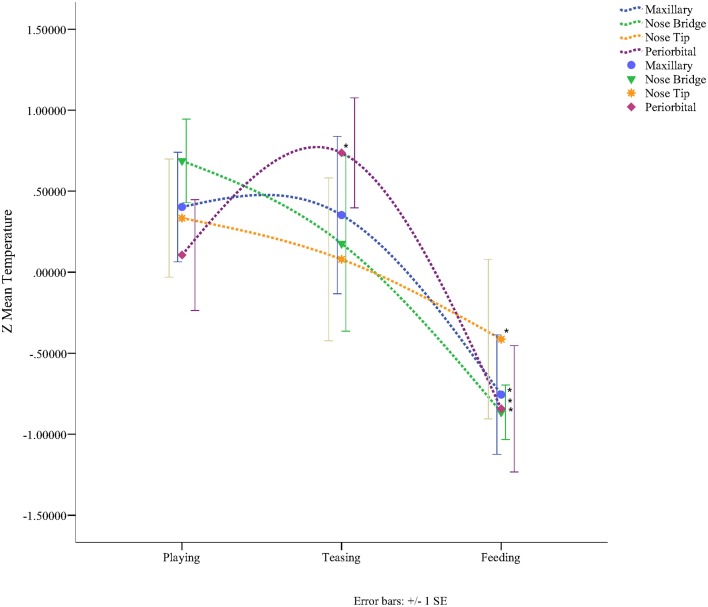
Graph representing the Z-mean temperature for each region of interest according to condition (^*^p **< 0.05)**.

In total four repeated measures ANOVA were conducted for each region of interest The results showed a significant main effect for condition on all regions of interest maxillary Wilk's Lambda = 0.03, *F*_(2, 3)_ = 49.82, *p* = 0.005, multivariate partial eta squared = 0.97, nose bridge Wilk's Lambda = 0.03, *F*_(2, 3)_ = 56.51, *p* = 0.004, multivariate partial eta squared = 0.97, nose tip Wilk's Lambda = 0.08, *F*_(2, 3)_ = 18.36, *p* = 0.02, multivariate partial eta squared = 0.92, orbital Wilk's Lambda = 0.12, *F*_(2, 3)_ = 11.03, *p* = 0.04, multivariate partial eta squared = 0.88. Within-condition contrasts yielded a significant temperature decrease for feeding compared to the preceding conditions [vs. (Teasing and Feeding)], on the maxillary area, the nose bridge and tip as well as the orbital region. Unlike other regions of interest the orbital region showed a significant temperature increase during the teasing phase only (see Tables 1, 2; Figures 3, 4).

**Table 1:** **Mean group values for 5 subjects according to region of interest**.

**Region**	**Condition**	**Mean**	***SD***
Maxillary	Playing	19.9949	0.81322
	Teasing	19.9412	1.16687
	Feeding	18.7499	0.88571
Nose bridge	Playing	22.3890	0.59751
	Teasing	21.8601	1.25224
	Feeding	20.7822	0.38889
Nose Tip	Playing	16.1118	0.77913
	Teasing	15.8687	1.07480
	Feeding	15.3976	1.05222
Peri-orbital	Playing	33.8925	1.11046
	Teasing	34.8078	1.10075
	Feeding	32.5157	1.26405

**Table 2:** Repeated measures ANOVA for within conditions contrasts for the 5 subjects (^*^p < 0.05; ^**^p **< 0.005) a computed using alpha = 0.05**.

**Region**	**Condition**	**Type III Sum of Squares**	**df**	**Mean Square**	***F***	***p***	**η^2^_*p*_**	**Observed Power**
Maxillary	Teasing vs.	0.014	1	0.014	0.044	0.845	0.011	0.53
	Feeding vs.	7.42	1	7.42	132.14	0.000**	0.971	1.00
Nose Bridge	Teasing vs.	1.41	1	1.41	2.441	0.193	0.379	0.227
	Feeding vs.	9.00	1	9.00	8.405	0.044*	0.678	0.591
Nose tip	Teasing vs.	0.31	1	0.31	1.361	0.308	0.254	0.149
	Feeding vs.	1.81	1	1.81	48.236	0.002**	0.923	0.999
Peri- Orbital	Teasing vs.	4.19	1	4.19	8.771	0.041*	0.687	0.609
	Feeding vs.	16.83	1	16.83	14.380	0.019*	0.782	0.806
**Region**	**Error (condition)**	**Type III Sum of Squares**	**df**	**Mean Square**				
Maxillary	Teasing vs.	1.32	4	0.330				
	Feeding vs.	0.225	4	0.056				
Nose Bridge	Teasing vs.	2.29	4	0.57				
	Feeding vs.	4.29	4	1.07				
Nose tip	Teasing vs.	0.91	4	0.22				
	Feeding vs.	0.15	4	0.04				
Peri- Orbital	Teasing vs.	1.91	4	0.48				
	Feeding vs.	4.68	4	1.17				

### Individual temperature in relation to condition

**Figure 5:**
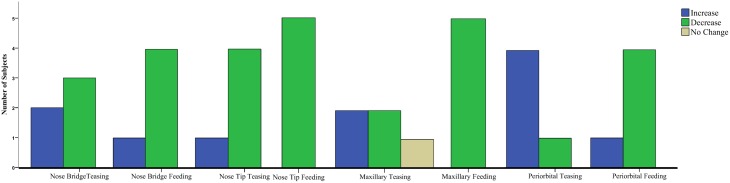
**Clustered Bar Chart representing the temperature tendency for all monkeys in each condition contrast for the Mann–Whitney** ***U*****-test**.

Results for the first contrast (Teasing vs. Playing) were in agreement with the group analyses. For the maxillary area 2 subjects had a significant increase and 2 a decrease, for the nose bridge 3 subjects showed a decrease compared to the condition, whereas for the nose tip 3 subjects had a decrease and 1 an increase. Finally for the peri-orbital region 4 subjects out of 5 had a significant increase during teasing compared to the previous condition. The second contrast [Feeding vs. preceding conditions (Playing and Teasing)] in its majority represented the group tendency. For the maxillary area all subjects had a significant decrease, for the nose bridge 4 out of 5 showed a decrease, whereas for the nose tip 5 out of 5. The orbital region showed a significant difference in 4 out of 5 subjects with 3 having a decrease and 1 an increase (see Table 3; Figure 5). The data used for the analyses can be downloaded from https://www.researchgate.net/profile/Stephanos_Ioannou.

**Table 3:** **Mann–Whitney** ***U***-tests, for all 5 individuals compared to preceding conditions (^*^p **< 0.05)**.

**Subjects**	**Region**	**Condition**	***U***	***P***	***Z***	***r***	***md***	***n***	**vs. *Md***	***n***
1-Spok	Maxill.	Teasing vs.	1385	0.317	−1	0.08	20.3	27	20.3	117
		Feeding vs.	80.5	0.000	−6.31	0.49*	19.2	17	20.3	144
	Nose B.	Teasing vs.	198	0.915	−0.106	0.01	22.6	7	22.5	58
		Feeding vs.	143.5	0.000	−4.47	0.49*	20.4	16	22.6	65
	Nose T.	Teasing vs.	531	0.07	−1.81	0.18	16.4	18	16.5	81
		Feeding vs.	272.0	0.000	−4.23	0.39*	15.45	16	16.4	99
	Orbital	Teasing vs.	0	0.000	−5.50	0.60*	35.35	12	33.4	70
		Feeding vs.	340.5	0.000	−4.26	0.41*	32.7	21	33.6	82
2-Drey	Maxill.	Teasing vs.	8	0.04	−2.05	0.49*	18.2	13	18.5	4
		Feeding vs.	10.50	0.002	−3.12	0.63*	17.6	7	18.3	17
	Nose B.	Teasing vs.	0	0.014	−2.47	0.29*	20.0	5	21.4	4
		Feeding vs.	16.00	0.825	−0.311	0.08	20.8	4	20.7	9
	Nose T.	Teasing vs.	0	0.00	−2.81	0.77*	14.0	9	14.75	4
		Feeding vs.	6.50	0.002	−3.10	0.69*	13.60	7	14.10	13
	Orbital	Teasing vs.	17	0.874	−0.159	0.04	34.3	9	34.4	4
		Feeding vs.	0	0.008	−2.67	0.67*	33.2	13	34.3	3
3-Tammy	Maxill.	Teasing vs.	0.500	0.018	−2.36	0.79*	21.3	5	20.5	4
		Feeding vs.	0.000	0.001	−3.19	0.82*	19.85	6	20.7	9
	Nose B.	Teasing vs.	4.00	0.135	−1.49	0.49	23.40	5	22.7	4
		Feeding vs.	0.000	0.003	−3.01	0.80*	20.40	5	22.9	9
	Nose T.	Teasing vs.	2.00	0.047	−1.98	0.66*	16.50	5	16.15	4
		Feeding vs.	4.50	0.008	−2.66	0.68*	15.90	6	16.40	9
	Orbital	Teasing vs.	0.000	0.010	−2.57	0.81*	36.00	4	34.65	6
		Feeding vs.	9.00	0.022	−2.28	0.57*	34.2	6	35.15	10
4-Minka	Maxill.	Teasing vs.	0.500	0.028	−2.191	0.77*	20.55	4	20.30	4
		Feeding vs.	0.000	0.001	−3.25	0.84*	18.9	7	20.4	8
	Nose B.	Teasing vs.	0.000	0.019	−2.33	0.82*	22.10	4	22.80	4
		Feeding vs.	8	0.020	−2.32	0.60*	21.30	7	22.5	8
	Nose T.	Teasing vs.	0.000	0.019	−2.35	0.83*	16.05	4	16.30	4
		Feeding vs.	0.000	0.001	−3.26	0.84*	15.5	7	16.15	8
	Orbital	Teasing vs.	0.000	0.019	−2.35	0.83*	35.15	4	34.75	4
		Feeding vs.	0.000	0.001	−3.25	0.84*	31.00	7	35.05	8
5-Hobo	Maxill.	Teasing vs.	8	0.004	−2.86	0.67*	19.6	10	20.55	8
		Feeding vs.	0.00	0.001	−3.36	0.70*	18.7	5	19.75	18
	Nose B.	Teasing vs.	0.00	0.003	−2.96	0.82*	21.35	8	22.4	5
		Feeding vs.	0.00	0.001	−3.22	0.76*	20.6	5	21.7	13
	Nose T.	Teasing vs.	7	0.013	−2.49	0.60*	16.2	12	16.5	5
		Feeding vs.	10	0.009	−2.59	0.74*	16.0	5	16.2	17
	Orbital	Teasing vs.	0.0	0.003	−2.94	0.85*	33.05	6	32.1	6
		Feeding vs.	0.0	0.003	−2.93	0.73*	31.35	4	32.6	12

### Behavioral analyses

It was tested if more rapid bodily movements could account for higher temperature values in the sample. The teasing data showed no significant temperature differences when the slow and rapid movements were compared (Mann–Whitney *U*, *N* = 3 + 3 subjects: maxillary: *U* = 4, *p* = 0.5; nose bridge: *U* = 3, *p* = 0.35; nose tip: *U* = 4, *p* = 0.5; orbital: *U* = 3, *p* = 0.35). The slow movement temperatures were higher for nose bridge (mean ± SD: 21.8 ± 0.6 and 21.7 ± 0.4, respectively) and orbital (34.0 ± 0.4 and 33.5 ± 0.7) and lower for maxillary (19.4 ± 0.4 and 19.6 ± 0.4) and nose tip (15.8 ± 0.4 and 16.3 ± 0.2) than the rapid movement temperatures. During play, the subjects showed only slow bodily movements.

## Discussion

The current study with the use of Functional Infrared Thermal Imaging systematically collected data on four regions of interest, recording significant physiological thermal changes across three different experimental conditions. This illustrates the potential benefits of this technique in exploring varying emotional states in non-human primates opening new avenues in the domain of behavioral neuroscience.

The simplicity of using fITI for physiological monitoring makes it particularly useful in experimental designs that resemble real life situations in which liberty of movement is essential. For example social emotions such as empathy are challenging to study virtually (e.g., films, photographs) as it is hard to substitute the social component with a stimulus presentation (Ebisch et al., 2013). The non-invasive nature of fITI makes it ideal for ecological experimental designs as well as for sensitive population groups such as individuals with disabilities and infants that cannot express their emotions verbally (Nakanishi and Imai-Matsumura, 2008). Its reliability has been examined simultaneously with gold standard physiological methods such as galvanic skin response (GSR) (Kuraoka and Nakamura, 2011; Pavlidis et al., 2012) laser Doppler flowmetry (Kistler et al., 1998) and polygraph testing (Pavlidis et al., 2002). Compared to GSR, Infrared Thermal Imaging provides equal or even better detection power between affective states. Variation of stimulus intensity is evident in facial temperature, whereas with GSR each stimulus, irrespective of intensity produces the same signal (Kuraoka and Nakamura, 2011). In addition to this drawback of hypersensitivity, GSR electrodes can loose conductivity over time (Levenson, 1988), signal changes can occur spontaneously during baseline (Laine et al., 2009). Nevertheless, the sluggish nature of facial temperature change makes infrared imaging not a good candidate for experiments that are concerned about the temporal latency of an emotional response. At the best-case scenario a significant temperature change can be observed within 10 s, whereas with GSR within 3 s (Kuraoka and Nakamura, 2011).

During the testing sessions, macaques showed stimulus-specific physiological activations. Temperature changes were evident on the peri-orbital region during teasing as well as on the upper lip, peri-orbital region and the nose (bridge and tip) during feeding. Compared to playing an increase in temperature was observed on the peri-orbital region during teasing in which 5 out of 5 subjects were intensely gazing at a mealworm/cricket that was quickly and repetitively passed in front of the mesh by the experimenter. Despite the fact that the results of this experimental condition are probably the result of increased blood perfusion in response to anger (Stemmler et al., 1991), it is also possible that prolonged activity of the main muscles surrounding the eyes (the corrugator, procerus, and orbicularis oculi) could have led to this effect. Subjects during this time were repetitively following a mealworm or a cricket that was initially approaching them and then quickly pulled away. This action resulted in rapid saccades by the subject that aimed at following the meal. Thus, retention of blood by the local musculature could have been observed supplied by the inferior palpebral and supraorbital arterial brunch of the facial artery. Similar results have been obtained in human studies on the peri-orbital region in experiments that used startle stimuli (Pavlidis et al., 2001) as well as mock interrogations (Pavlidis et al., 2002) after fake crime scenes. Lastly, whereas the temperature of the maxillary area during teasing did not show any consistency among subjects, the nose bridge and tip did show a tendency for a decrease. The reason why this did not reach statistical significance could be related to the time-length that each monkey spent in the teasing condition. On the contrary the reason why the peri-orbital region managed to make a leap in temperature is not only because is innervated by large facial arteries but also by the fact that the skin around the ocular cavity is rather thin compared other region of interest. Thus, temperature changes and inter-frame variability are more pronounced.

Feeding was predominantly associated with a temperature decrease on all regions of interest. In this phase of the experiment monkeys no longer had to follow a moving meal but they are being hand fed by the experimenter. Nevertheless, although the temperature of the peri-orbital region is decreasing, as no sustained muscular activity by the eye musculature is required during this experimental phase, the same phenomenon is observed on the rest of the regions of interest. One would expect a rise in temperature during feeding, as the monkeys are no longer engaged in a stressful task. Nevertheless, the biological signs that they exhibit suggest otherwise and are in agreement with studies that exposed subjects to fearful stimuli (Nakayama et al., 2005; Kuraoka and Nakamura, 2011). Feeding was perhaps inducing some level of stress (Waitt and Buchanan-Smith, 2001). Both fear and anger are associated with increased α-adrenergic activity, which is responsible for the evacuation of heat from the surface of the skin as a result of vascular constriction (Kreibig, 2010). Thermal imaging experiments showed that the nose provides the most reliable indicator of negative states due to large presence of arteriovenous anastomoses (Bergersen, 1993) and makes sympathetic vasoconstrictive effects, accompanying temperature drops, more evident (Nakayama et al., 2005; Kuraoka and Nakamura, 2011; Ioannou et al., 2013). For example, in the case of Vianna and Carrive (2005), whereas body and head temperature in fear conditioned rats was rising, on the paws, tail and nose it was showing the opposite pattern. This decrease in temperature occurs mainly through smooth muscles in arterioles and arteriovenous anastomoses in distal body regions guarding against excessive hemorrhage in case of an injury. The reason why is located mainly in extremities is because that these regions have extensive contact with the environment (Hales, 1985). Moreover, it is important to note that the decrease in temperature on distal body regions persists despite increases in heart rate. Finally the decrease on the maxillary area is related to the activation of perspiration pores as a result of sympathetic arousal (Shastri et al., 2009; Pavlidis et al., 2012). Emotional sweating has been proposed to increase elasticity and reduce friction of skin regions that have increased contact with the environment and external objects (Porges, 1992; Kamei et al., 1998; Vetrugno et al., 2003).

Non-human primates seem to share a strong resemblance with humans in the way that they respond to stimuli of affective nature. Not only they share a similar facial arterial anatomy (see Figure 1 in Ioannou et al., 2014a) but when it comes to the thermal responses in relation to fear, threat or distress similar patterns arise. In humans temperature decrease on the nose has been observed in response to startles (Naemura et al., 1993), during toy mishaps (Ioannou et al., 2013), as well as in cases of increased mental workload (Kang et al., 2006; Calvin and Duffy, 2007). In monkeys we have only two occasions where a phenomenon as such happened and this was in response to fear (Nakayama et al., 2005; Kuraoka and Nakamura, 2011). The upper lip in humans has shown a decrease in temperature and an increase in the count of perspiration pores under stress (Pavlidis et al., 2012) as well as startles (Shastri et al., 2012). The current study provides the only indication to date that a decrease on the upper lip can also occur in rhesus monkeys under varying affective states. On the other hand the peri-orbital region has previously shown a tendency for an increase in response to a threatening person although it did not reach statistical significance (Nakayama et al., 2005). Temperature increase on the peri-orbital region was evident in humans not only during startles and mock interrogations but also in a rather demanding social context where a unified facial increase was observed (Ioannou et al., 2014b).

Body temperature is directly linked with physiology as changes in blood flow affect the emitted thermal print (Ioannou et al., 2014a). Physiological changes have been related to particular emotional conditions (Kreibig, 2010) and in the case of thermal imaging temperature seems to respond in particular ways in different body parts. So far functional infrared thermal imaging (fITI) has based the majority of observation on the thermal print of the face. The rich underlying vasculature makes the face an ideal candidate for monitoring peripheral physiology as changes in heart rate, muscular adaptation and epinephrine release in the blood stream are depicted in specific facial regions with unique temperature element (Ioannou et al., 2014a). Negative emotions such as fear (Nakayama et al., 2005) and guilt (Ioannou et al., 2013) have been associated with a decrease in nose temperature, a phenomenon associated with subcutaneous vascular constriction, evident on the extremities of the body [(nose, ears, fingers in humans) and (paws, ears, tail in animals)]. On the contrary social interaction (Ioannou et al., 2014b) and sexual arousal (Hahn et al., 2012) have been associated with a temperature increase on the majority of facial regions probably signaling the felt emotion. Moreover, in occasions of distress (such as mental workload or startle) perspiration pores increase (Pavlidis et al., 2012), leading to a temperature decrease on the upper lip, whereas the peri-orbital region of the ocular cavity shows the opposite pattern, an increase in temperature (Pavlidis et al., 2001). Authors suggest that the local musculature that surrounds the eyes is responsible for the temperature increase of the peri-orbital region and this happens to facilitate rapid saccades in case of a perceived threat. The maxillary area (or the upper lip) although not among the regions of affective sweating (such as the axillae, palms, and soles of the feet) shows a positive correlation with the fingers in response to startles. Thus, inferences can be made of emotional arousal by observing the thermal signature of the upper lip (Shastri et al., 2009). Conclusively the face can be used to observe holistic autonomic responding deriving from the endocrine system to nerve impulses of the vagus to the heart. Autonomic adaptation carries its own physiological thermal print and by harnessing the power given by homeostatic balance distinctions can be made between arousal and parasympathetic restoration.

The reason why affective research in monkeys is limited is because of the methodological roadblocks that one has to face in order to carry out successfully the experiment but also to record biological signs. Thermal imaging provides an alternative for physiological research that could enhance and support behavioral observations. Nonetheless manual extraction of thermal data is a laborious process but currently no tracking algorithms have been applied to moving monkeys in order to make analyses of the data more efficient. Experimental protocols should be within realistic goals and need to keep the subjects interest in the task, as it is rather difficult to maintain the animal's attention uninterrupted throughout the experiment. To tackle such a problem one should consider acquiring data of the same subject for multiple days within the same context and for a very short period of time. Future studies should consider including other primate species either in enclosures or even in the wild. Finally it would be worth to explore other experimental paradigms that do not involve a negative component such as fear but deal with the social elements of a primate's life such as grooming as well as disputes.

Overall the current study showed varying emotional states in a group of macaques non-invasively. Thermal imaging has successfully collected data across conditions without any restriction of the subject, and represents a reliable physiological tool that can grasp the physiological responses of subjects enabling applications in variety of experimental settings while enriching behavioral observations.

### Conflict of interest statement

The authors declare that the research was conducted in the absence of any commercial or financial relationships that could be construed as a potential conflict of interest.
